# Differential roles of Stella in the modulation of DNA methylation during oocyte and zygotic development

**DOI:** 10.1038/s41421-019-0081-2

**Published:** 2019-01-29

**Authors:** Longsen Han, Chao Ren, Jun Zhang, Wenjie Shu, Qiang Wang

**Affiliations:** 1 State Key Laboratory of Reproductive Medicine, Nanjing Medical University, Nanjing, Jiangsu 211166 China; 2 Department of Biotechnology, Beijing Institute of Radiation Medicine, Beijing, 100850 China

**Keywords:** Epigenetic memory, Chromosomes

Dear Editor,

Stella (also known as PGC7 or Dppa3) was identified as a highly expressed protein in primordial germ cells (PGC). Expression of Stella is maintained throughout oocyte maturation and persists into the preimplantation embryos^1^. *Stella* knockout females display a strongly reduced fertility due to the impaired developmental competence of early embryos^2^. The potential involvement of Stella in embryogenesis and generation of induced pluripotent stem (iPS) cells has been examined^3,4^. However, the effects of Stella on oocyte development, especially at cellular and molecular level, remain unknown. To do this, we generated Stella mutant mice using the CRISPR/Cas9 system (Supplementary Fig. S1a). Mice carrying homozygous mutations were born alive in a knockout line containing 153-bp deletion (Stella^Δ^). We confirmed the successful deletion of Stella in the mutant mice by western blot analysis (Supplementary Fig. S1b). Stella^Δ^ females were infertile when crossed with mutant males despite the ovulation of eggs with normal appearance (Supplementary Fig. S1c-e). After fertilization, Stella^Δ^ embryos are compromised in the preimplantation development and rarely reach the blastocyst stage (Supplementary Fig. S1f-g), consistent with published data^2^. It is worth noting that this truncated protein was absent in the nucleus, but was still detectable in the cytoplasm of Stella^Δ^ zygotes based on immunostaining (Supplementary Fig. S2, arrows). Recently, Shin et al., revealed that maternal Stella is partially cleaved by the ubiquitin-proteasome system and an N-terminal fragment remains in the cytoplasm where it participates in vesicular trafficking^5^. Therefore, our mutant mouse model may provide novel insights into Stella function compared to the conventional knockout mice reported previously^6,7^.

Given that Stella modulates the epigenetic asymmetry in zygotes, we asked whether Stella is also involved in the establishment of DNA methylation during oogenesis. To address this question, ovulated oocytes from Stella^Δ^ and wild-type (WT) mice were isolated, and then base-resolution methylomes were generated using the bisulfite sequencing (BS-Seq) method for small samples (Fig. 1a). We found that, in WT oocytes, the global DNA methylation level was ~38%, as expected^8^. However, in Stella^Δ^ oocytes, the average methylation level was dramatically increased to ~68% (Fig. 1b–c). This extensive elevation of DNA methylation was observed across all genomic features examined, such as promoter, untranslated region (UTR), CpG island (CGI), intron, exon, as well as the major repetitive-elements (Fig. 1d–g; Supplementary Fig. S3). Such a pattern indicates that the changes in DNA methylation of Stella^Δ^ oocytes are in general universal throughout the entire genome. To gain a better understanding of the altered methylation landscape, we also conducted a search for differentially methylated regions (DMRs) between WT and Stella^Δ^ oocytes. In total, 21,036 DMRs were identified, of which 20,998 were hypermethylated (hyper-DMRs; 99.8%) and only 38 were hypomethylated (hypo-DMRs; 0.2%) (Fig. 1h; Supplementary Table S1), showing a predominance of hyper-DMRs. In the female germline, de novo methylation takes place during the postnatal growth phase of oocytes. Stella was shown to be able to inhibit recruitment of the DNA methyltransferase, DNMT1, through the binding of UHRF1^9^, which might be the critical pathway mediating the effects of Stella on methylation landscape in oocytes. Together, our findings clearly suggest that Stella is a novel and essential factor preventing excessive DNA methylation during oocyte development.

**Fig. 1:**
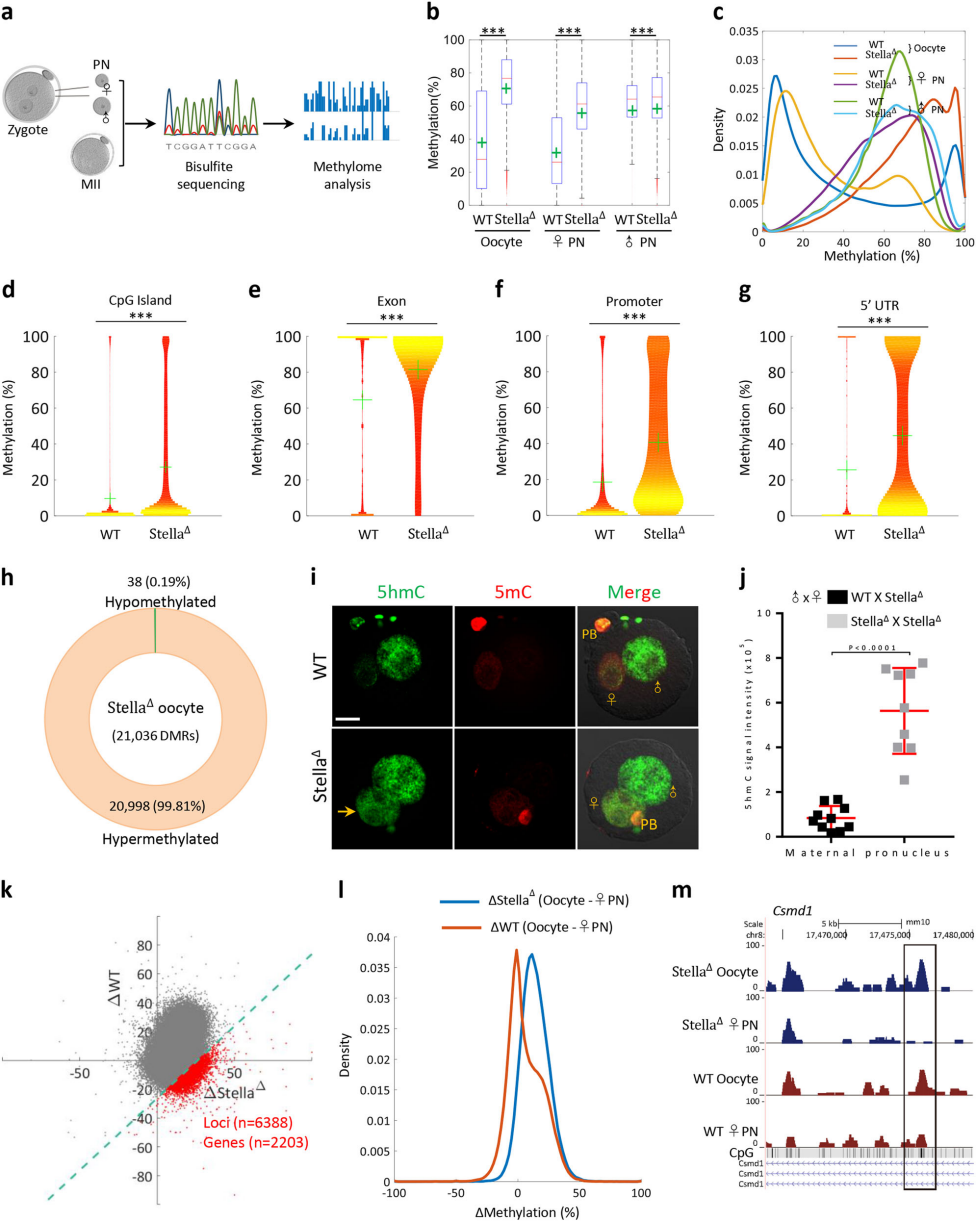
Differing roles of Stella in the control of DNA methylation during oocyte and zygotic development. **a** Diagram illustrating the BS-seq procedure for genome-wide methylation analysis. Individual parental pronuclei and ovulated oocytes were collected, and DNA was bisulfite converted, followed by library preparation and high-throughput sequencing. **b** Distribution of the average methylation level across 20-kb windows in oocyte, female pronucleus (♀PN), male pronucleus(♂PN) from Stella^Δ^ and WT mice. Boxplot illustrates the median (red bar), mean (green cross), 25/75 percentage range (box), maximum and minimum (whiskers), and extreme values (red dots outside box). **c** Density plot of the average methylation level across 20-kb windows in oocyte, ♀PN, ♂PN from Stella^Δ^ and WT mice. **d**–**g** Violin plots show the methylation levels for four genomic features in oocytes from Stella^Δ^ and WT mice. The green cross indicates the mean methylation levels. Bootstrap test was used to for statistical analysis. **h** Total number of DMRs identified between oocyte from Stella^Δ^ and WT mice. The proportions of hyper- and hypo-DMRs are presented in circular plot. **i** PN4 zygotes from WT and Stella^Δ^ mice were stained with anti-5mC (red) and anti-5hmC (green) antibodies. Arrows indicate the gain of 5hmC in the maternal PN of Stella^Δ^ zygotes. PB, polar body. Scale bar, 20 μm. **j** Quantification of 5hmC fluorescence intensity in female pronuclei of zygotes. Each data point represents one maternal PN in zygotes (*n* = 10 for each group). A Student’s *t*-test was used for statistical analysis. **k** Scatter plot illustrates the difference in DNA methylation level across 20-kb windows in the groups as indicated. *X*-axis represents the difference in DNA methylation level between oocyte and ♀PN from Stella^Δ^ mice, and *Y*-axis represents the difference in DNA methylation level between oocyte and ♀PN from WT mice. The red dots denote the 20-kb windows with large difference (<5%) in DNA methylation level in Stella^Δ^ group compared with WT group. Green line $\left( {X - Y = 26.31} \right)$ indicates the threshold corresponding to 5%. **l** Density plot of the difference in average DNA methylation level across 20-kb windows between oocyte and ♀PN from Stella^Δ^ and WT mice. **m** Screenshot of *Csmd1* gene, as an example of a gene whose methylation is protected by Stella following fertilization

To track the effects of Stella on the DNA methylation of parental genome, individual female and male pronuclei of late-stage zygotes were isolated separately for genome-wide profiling (Fig. 1a). Limited change was observed in the methylation levels of maternal DNA between oocytes and zygotes from WT mice, as expected^8^. However, maternal DNA methylome is markedly demethylated from oocytes (68%) to zygotes (55%) in Stella^Δ^ mice (Fig. 1b–c). In support of this, we found a significant increase in 5hmC signal in the female pronuclei of Stella^Δ^ zygotes; whereas female pronuclei in WT zygotes showed a much less intense 5hmC staining (Fig. 1i–j). These results strongly indicate the active demethylation of maternal genome during Stella^Δ^ zygote development, supportive of the model proposed by Wang et al., Nakamura et al., and Armouroux et al.^8,10^. TET3 is a critical dioxygenase that catalyzes conversion of 5mC to 5hmC in the paternal genome, while Stella could block TET3 activity to maintain DNA methylation of the maternal DNA^4,7^. Heavy demethylation of maternal genome in Stella^Δ^ zygotes was likely due to the TET3 gaining access to female pronuclei. Next, in order to search for those potential loci and genes whose methylation is protected by Stella, we analyzed the difference in DNA methylation level of 20 kb windows between oocyte and maternal pronuclei from WT and Stella^Δ^ mice, respectively. Totally, 6388 genomic loci and 2203 genes were identified (Fig. 1k–l; Supplementary Table S2), such as the development-related genes *Csmd1* (Fig. 1m), *Zdhhc6*, and *Erbb4* (Supplementary Fig. S4). Gene ontology (GO) analysis further indicates that these genes are enriched in the pathways that play important roles in nervous system and metabolic process (Supplementary Fig. S4). Interestingly, compared to WT zygotes, the average methylation level of paternal genome in Stella^Δ^ zygote was increased significantly, although not dramatically (Fig. 1b–c). More assays are needed to clarify this issue. Cumulatively, our findings suggest that Stella participates in the DNA methylation maintenance of maternal genome during mouse zygotic development.

On the other hand, we noticed that, although the DNA demethylation has occurred, the average methylation level of female pronuclei was still elevated in Stella^Δ^ zygotes when compared to WT zygotes (Fig. 1b–c). This observation prompted us to propose that such a high level of maternal DNA methylation in Stella^Δ^ zygotes was likely originated from the global hypermethylation in oocytes. To test this possibility, we evaluated the contribution of different genomic features to the DNA hypermethylation in Stella^Δ^ oocytes and zygotes. Gain of methylation was detected in all elements of Stella^Δ^ oocytes, with the largest proportion contributed by intergenic region (48%; Supplementary Fig. S5a-b). Maternal genome of Stella^Δ^ zygotes displayed the similar pattern compared with their WT counterparts (Supplementary Fig. S5a-b). Of note, the extent of demethylation of distinct elements was almost identical between oocytes and female pronuclei in either WT or Stella^Δ^ mice, as evidenced by similar methylation patterns (Supplementary Fig. S5b-c). This observation indicates that Stella has no preference for the specific genomic regions when protecting maternal DNA against demethylation in zygotes. Moreover, we found that 91% of genes with hyper-DMR (621/680) identified in maternal genome of Stella^Δ^ zygotes were indeed inherited from their oocytes (Supplementary Fig. S6). Altogether, these data suggest that global hypermethylation across the oocyte methylome in Stella^Δ^ mice results in the higher level of DNA methylation in female pronuclei than that in WT mice. Considering that BS-Seq measures the sum of 5mC and 5hmC, and the strong 5hmC signals were detectable in Stella^Δ^ female pronuclei, more dramatic DNA demethylation perhaps occurred during the transition from oocytes to zygote in Stella^Δ^ mice.

In summary, by constructing a Stella mutant mouse model, we identified Stella as a novel factor essential for preventing excessive DNA methylation during oogenesis. Following fertilization, Stella participates in the maintenance of maternal genome methylation during zygotic development. After we submitted this manuscript, Li et al. reported the function of Stella in safeguarding the oocyte methylome^11^, further supporting our conclusion. Our work also provides a comprehensive atlas at the genome-wide scale of the DNA methylation landscape in oocytes and zygotes from Stella^Δ^ mice, which offers new insights into the Stella function in epigenetic control.

## Supplementary information

Supplemental Information

Supplemental Table S1

Supplemental Table S2

## Electronic supplementary material

**Supplementary Information** accompanies the paper at (10.1038/s41421-019-0081-2).
