# Supplementary material for: Differential roles of Stella in the modulation of DNA methylation during oocyte and zygotic development
Source: Cell Discov. 2019 Jan 29;5:9. doi: 10.1038/s41421-019-0081-2 (PMC6349861; doi:10.1038/s41421-019-0081-2)
Supplement: Supplementary file 1 — Supplemental Information [file 41421_2019_81_MOESM1_ESM.pdf]

Supplemental Materials and Methods

Mice

All animal experiments followed the rules and guidelines of the local animal ethical committee and the Animal Care and Use Committee of Nanjing Medical University. All study protocols were approved by the Animal Care and Use Committee of Nanjing Medical University. Cas9 plasmid Pst1374-Cas9-N-NLS-Flag-linker and expression vectors for sgRNAs were provided by Professor JunZhang (Nanjing Medical University). Exon2 of the Stella gene was targeted by two sgRNAs. Oligos sequences are as follows: sgRNA1-up: taggTGTCAAGCGGTCCGCACGC; sgRNA1-down: aaac GCGTGCGGACCGCTTGACA; sgRNA2-up: taggAACAAGTCTTCTCATCTTTG; sgRNA2-down: aaacCAAAGATGAGAAGACTTGTT. mRNAs were obtained by *in vitro* transcription as described previously [1]. Cas9 mRNA/sgRNA injection to zygotes obtained by mating of C57BL/6J males with superovulated C57BL/6J females was also performed as described previously [1]. Male mice with a large deletion were chosen as founder animals, and backcrossed to establish a germline transmission line. Heterozygous deletion mice were intercrossed to obtain homozygous mice (Stella$^{\Delta}$). Genotyping primers are listed below: F: AACCCACAGTAAAGTAGCG; R: GGCTGGAGTTGCTCTTAG.

Fertility test: To test the fecundity, five individually housed WT and Stella$^{\Delta}$ mice were mated with Stella$^{\Delta}$ male mouse validated to be fertile at the age of 8 weeks. Cages were examined twice a week and the number of pups was recorded.

Preimplantation embryo development analysis: Mice received an injection of hCG 2 days after PMSG priming and were then mated overnight with males of proven fertility. Zygotes were obtained by flushing the oviducts 24 hours after the hCG injection, and cultured in KSOM medium (Millipore) for subsequent analysis.

Protein immunoblot

A total of 100 MII oocytes were lysed at 95C for 5 min in 2xSDS buffer. Protein extracts were separated using SDS-PAGE and electrically transferred to polyvinylidene fluoride membranes. After blocked in 5% low-fat milk for 1h, the membranes were incubated at 4C overnight with rabbit anti-Stella antibody (1:1,000; Abcam, ab19878) or mouse anti-β-actin antibody (1:2,000; Sigma, A5441). After three washes in PBST and

incubation with horseradish peroxidase (HRP)-conjugated secondary antibodies (1:5,000; Thermo Fisher Scientific) at room temperature for 1 hour, signals were detected using Pierce ECL western blotting substrate according to the manufacturer's instructions.

Immunofluorescence

5mC/5hmC staining: The 5mC/5hmC staining was carried out as we described previously [2]. In brief, the zona pellucida was dissolved by incubation with Acidic Tyrode's Solution (Sigma-Aldrich, St. Louis, MO, US), and then zygotes were fixed with 3.7% paraformaldehyde for 20 min. After incubated in 4M HCl solution for 10 min, samples were neutralized in Tris–HCl (pH 8.0) for 10 min. Following a second fixation with 3.7% paraformaldehyde, zygotes were treated with 0.2% Triton X-100 for 10 min. After overnight blocking at 4°C in 1% BSA, samples were stained overnight at 4°C with rabbit polyclonal anti-5hmC antibody (1:500; Active Motif, CA, Cat#: 39769) and mouse monoclonal anti-5mC antibody (1:500; Calbiochem, Cat#: NA81). The cells were washed and incubated with Alexa-Flour 488- or 555-conjugated secondary antibodies (1:150; Molecular Probes) for 4 hours at room temperature. Samples were mounted on slides with a drop of anti-fade medium (Vectashield, Burlingame, USA) and then examined under a laser-scanning confocal microscope (LSM710; Zeiss, Germany).

Stella staining: Zygotes were fixed with 4% paraformaldehyde for 30 min, permeabilized with 0.5% Triton X-100 for 20 min, blocked for 1 hour in 1% BSA-supplemented PBS at room temperature, and then incubated overnight at 4°C with anti-Stella antibody (1:500; Abcam, ab19878). Samples were washed three times in 0.1% Tween-20 in PBS containing 1% BSA before application of secondary antibodies. DNA was counterstained with propidium iodide. After three washes in PBST, samples were mounted on slides with a drop of anti-fade medium and then examined under a laser-scanning confocal microscope.

Sample preparation and bisulfite sequencing

The Narishige micromanipulator was equipped with a Piezo unit (PrimeTech) for zona drilling and breaking plasma membranes. The female and male pronuclei were isolated

according to their size and the distance from the second polar body. The oocytes and isolated pronuclei were subjected to DNA methylome as described previously [3] with minor modifications. Briefly, samples were lysed in lysis buffer (10 mM Tris-HCl pH 7.4 and 2% SDS) with 0.5 µl protease K for 1 h at 37 °C. Bisulfite conversion was performed on cell lysates using the EZ DNA Methylation-Gold Kit (Zymo Research) according to the manufacturer's instructions. The converted DNA was processed for library construction, and the quality and quantity of the purified library assessed using Agilent Bioanalyzer and StepOnePlus Real-Time PCR System (Applied Biosystems). Finally, libraries were prepared for 125-bp paired-end sequencing on a HiSeq2500.

Data analysis of zygote methylome

Raw reads are processed using Trim Galore (http://www.bioinformatics.babraham.ac.uk/projects/trim_galore/) to remove adapters and low-quality reads. Processed reads are mapped to the mouse genome assembly (GRCm38) using Bismark[4] with parameters '*single-end mode, options –non-directional,-bam,--bowtie2*'. Methylation levels of CpG sites are extracted by toolkits provided by Bismark. To assess the distribution of methylation level of whole genome, the genome is divided into 20 kb windows and average methylation level is calculated. Windows without valid CpG sites are removed from analysis. CpG islands (CGIs) and CGI shores were extracted from the CpG islands track of the UCSC Genome Browser[5]. Promoters were defined as the region 2kb upstream from TSSs (transcriptional start sites) annotated by UCSC RefSeq[6]. Exons, introns, UTRs, and intergenic regions were defined according to annotation of UCSC RefSeq. All repeat elements were extracted from the Simple Repeatstrack of the UCSC Genome Browser[5].

The average methylation level is defined as $\frac{\sum M(CpG)}{N}$, in which $M(CpG_i)$ denotes the methylation level of CpG sites and N denotes the total number of sequenced CpG sites. To quantify the contribution from genomic region i to average methylation level of whole genome, we defined average methylation contribution as $Con_i = \frac{\sum(M(CpG_i))}{N_i} * \frac{Ni}{N} = \frac{\sum(M(CpG_i))}{N}$. $M(CpG_i)$ denotes the methylation level of CpG sites in genomic region i and N_i denotes the total number of sequenced CpG sites in genomic region

i. The contribution percentage is defined as $\frac{Con_i}{Average(M)}$. The Con_i denotes the contribution of genomic region i and $Average(M)$ denotes the average methylation level of whole genome.

Metilene program[7] is used to identify differential methylated regions (DMRs) between groups with default parameters. Gene Ontology (GO) analysis of genes was performed using DAVID[8] (http://david.abcc.ncifcrf.gov). GO terms with adjusted *P*-value less than 0.05 were determined to be statistically significant.

References

1 Hu X, Shen B, Liao S *et al.* Gene knockout of Zmym3 in mice arrests spermatogenesis at meiotic metaphase with defects in spindle assembly checkpoint. *Cell death & disease* 2017; **8**:e2910.
2 Han L, Ren C, Li L *et al.* Embryonic defects induced by maternal obesity in mice derive from Stella insufficiency in oocytes. *Nature genetics* 2018; **50**:432-442.
3 Smallwood SA, Lee HJ, Angermueller C *et al.* Single-cell genome-wide bisulfite sequencing for assessing epigenetic heterogeneity. *Nature methods* 2014; **11**:817-820.
4 Krueger F, Andrews SR. Bismark: a flexible aligner and methylation caller for Bisulfite-Seq applications. *Bioinformatics* 2011; **27**:1571-1572.
5 Speir ML, Zweig AS, Rosenbloom KR *et al.* The UCSC Genome Browser database: 2016 update. *Nucleic acids research* 2016; **44**:D717-725.
6 O'Leary NA, Wright MW, Brister JR *et al.* Reference sequence (RefSeq) database at NCBI: current status, taxonomic expansion, and functional annotation. *Nucleic acids research* 2016; **44**:D733-745.
7 Juhling F, Kretzmer H, Bernhart SH, Otto C, Stadler PF, Hoffmann S. metilene: fast and sensitive calling of differentially methylated regions from bisulfite sequencing data. *Genome research* 2016; **26**:256-262.
8 Huang da W, Sherman BT, Lempicki RA. Systematic and integrative analysis of large gene lists using DAVID bioinformatics resources. *Nature protocols* 2009; **4**:44-57.

Supplementary Figures

Supplementary Fig. S1.

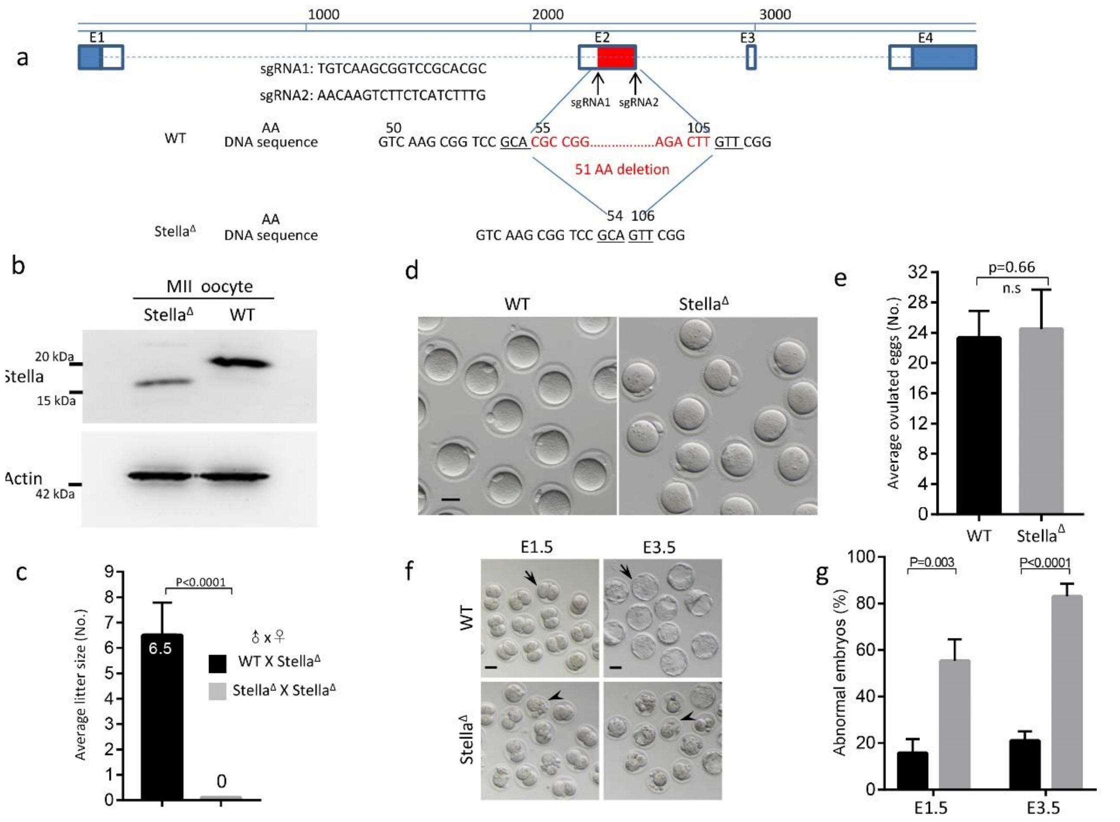

Stella is required for early embryo development. (a) Mouse Stella gene structure and gene-targeting strategy, including sgRNAs target position and sequences, and founder mouse deletion region. (b) Western blot analysis confirms the absence of Stella full length protein in oocytes. A smaller band was detected due to a 51-AA deletion. (c) Fertility test of WT and Stella$^{\Delta}$ female mice (n=5 for each group) by crossing with Stella$^{\Delta}$ males. Data are expressed as mean ± SD. (d) Representative bright-field images of MII oocytes recovered from WT and Stella$^{\Delta}$ mice. (e) Quantification of ovulated oocytes from WT and Stella$^{\Delta}$ mice. (f) Representative bright-field images of E1.5 and E3.5 embryos derived from WT and Stella$^{\Delta}$ females. Arrows indicate the examples with normal morphology; arrowheads indicate the abnormal embryos, such as cytoplasmic fragmentation and developmental arrest. (g) Quantification of abnormal embryos from WT and Stella$^{\Delta}$ mice (n=90 for each group). Data are expressed as mean ± SD from three independent experiments. A Student's *t* test was used for statistical analysis. Scale bar, 50 µm. n.s., not significant.

Supplementary Fig. S2.

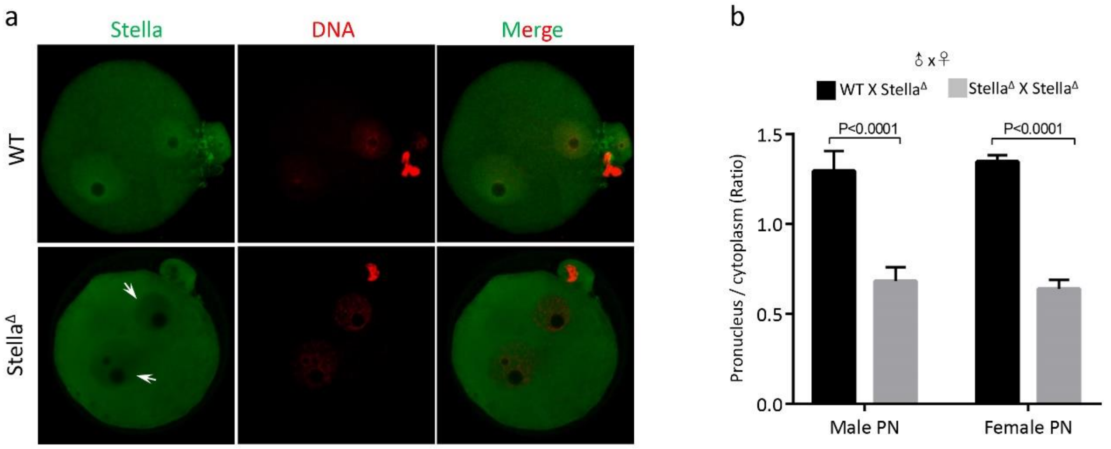

Mutant Stella is absent in the parental pronuclei of Stella$^{\Delta}$ zygotes. (a) Zygotes were immunostained with Stella antibody (green) and counterstained with propidium iodide (red) to visualize DNA. Stella staining was observed in both pronuclei and cytoplasm of normal zygotes, whereas Stella signals were absent in the parental pronuclei of Stella$^{\Delta/\Delta}$ zygotes (arrows). (b) Quantification of Stella intensity as a ratio between signal from the pronuclei relative to the signal from cytoplasm in zygotes as presented in (a) (n=15 for each group). Data are expressed as mean ± SD from three independent experiments. A Student's *t* test was used for statistical analysis. Scale bar, 20 µm.

Supplementary Fig. S3.

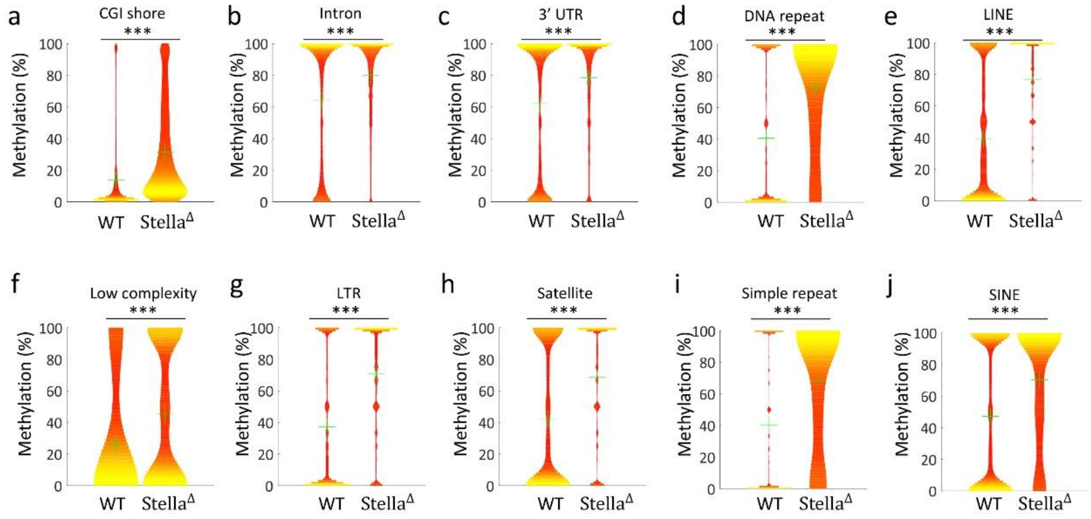

Violin plots showing the methylation levels for different genomic elements in oocytes from Stella$^{\Delta}$ and WT mice. The green cross denotes the mean methylation levels.

Bootstrap test was used to calculate the significance of difference.

Supplementary Fig. S4.

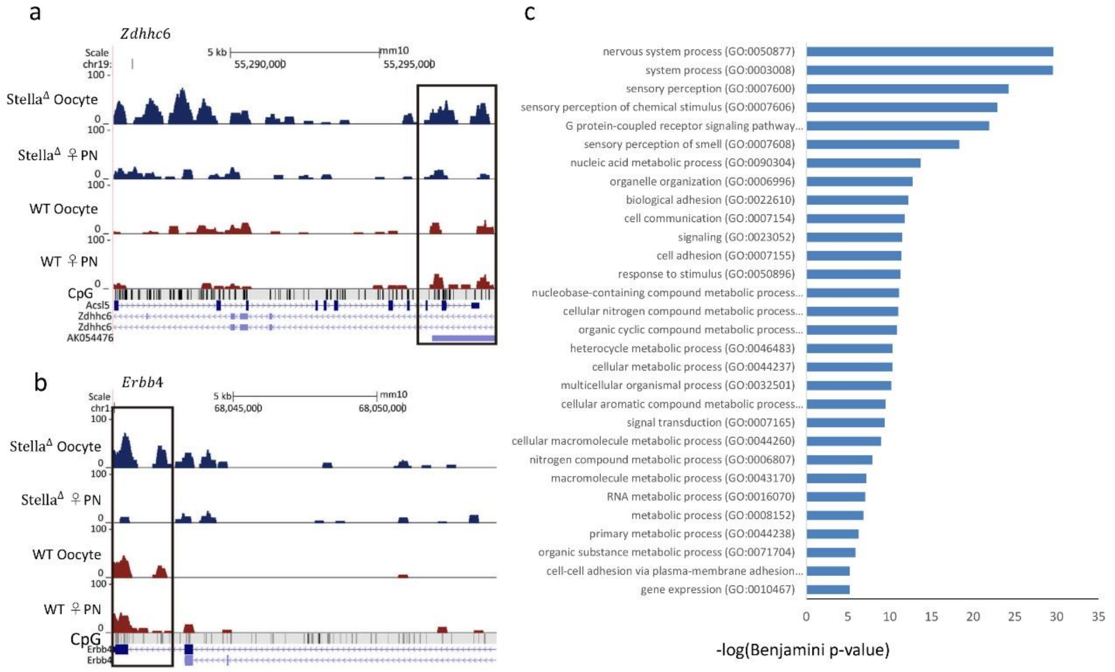

Gene ontology analysis for the potential genes protected from demethylation by Stella. The difference in methylation level is defined over 20-kb windows across the genome in oocyte and female pronucleus from WT and Stella$^{\Delta}$ mice. GO terms with Benjamin score < 0.05 are shown in the graph.

Supplementary Fig. S5.

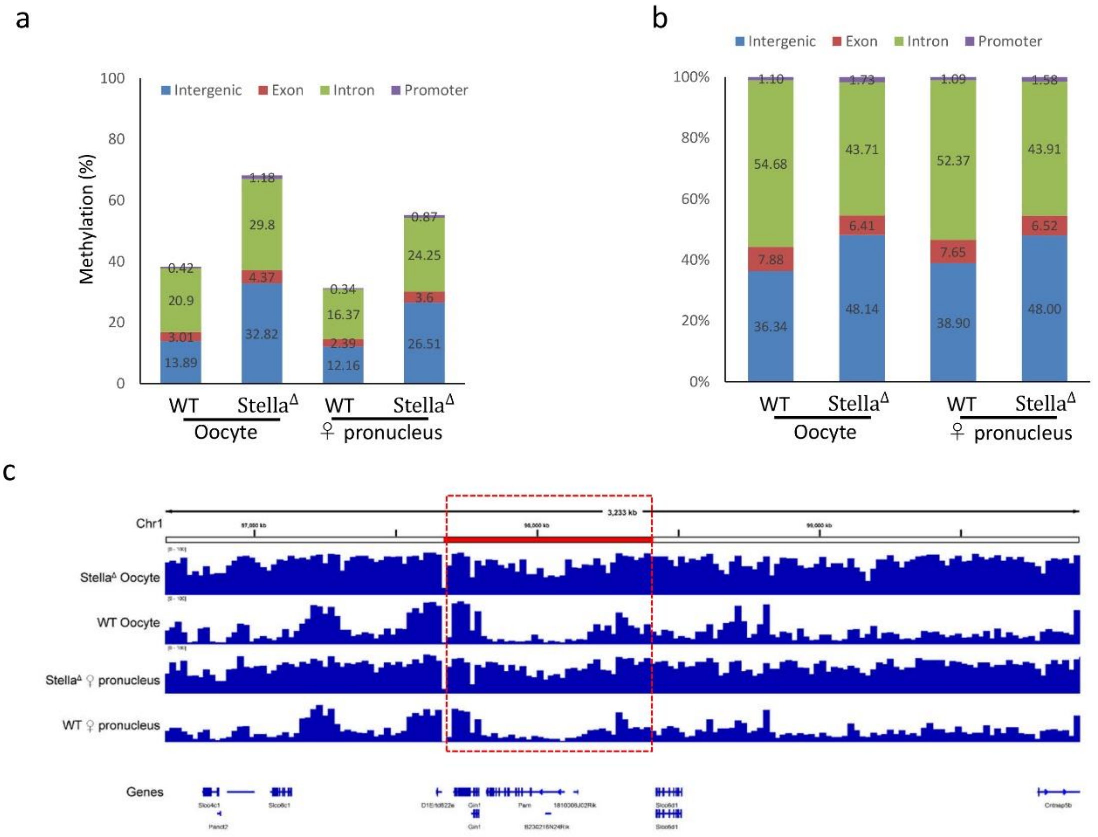

Histogram showing the contribution of different genomic regions to the hypermethylation in Stella$^{\Delta}$ oocytes and zygotes. (a) The average methylation contribution of each genomic element in oocytes and female pronucleus from WT and Stella$^{\Delta}$ mice. (b) The contribution percentages of each genomic element to the methylation extent in oocytes and female pronucleus from WT and Stella$^{\Delta}$ mice. (c) An example shows a representative hypermethylated region in female pronucleus of Stella$^{\Delta}$ zygotes was inherited from their oocytes. The average methylation contribution of genomic regions and their contribution percentages are defined in Materials and Methods section.

Supplementary Fig. S6.

a

Stella$^{\Delta}$ *vs*. WT	Totoal DMRs	Hyper DMRs	Hypo DMRs	Genes with hyper-DMR	Genes with hypo-DMR
Oocyte	21,036	20,998	38	3,216	20
Female PN	1,928	1,910	18	680	11

b

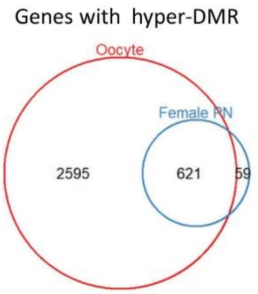

c

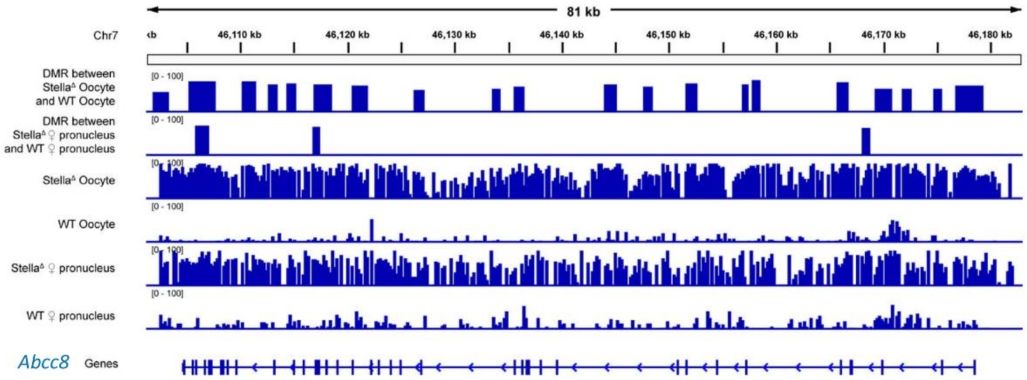

Analysis of the DMRs identified between oocyte and female pronucleus from WT and Stella$^{\Delta}$ mice. (a-b) Venn plot and Table summarize the overlapping DMRs between oocytes and pronucleus. (c) An example illustrating the methylation level of gene *Abcc8*. The height is corresponding to the difference in DNA methylation level between oocyte and female pronucleus.
